# Acute Central Retinal Vein Occlusion Secondary to Reactive Thrombocytosis after Splenectomy

**DOI:** 10.1155/2014/930843

**Published:** 2014-09-08

**Authors:** Nursen Oncel Acir, Mehmet Borazan, Zeynep Dadaci

**Affiliations:** Department of Ophthalmology, Mevlana University School of Medicine, (Aksinne M., Esmetas S. No. 16) Meram, 42100 Konya, Turkey

## Abstract

The diagnosis and treatment of central retinal vein occlusion was reported in a young patient. Central retinal vein occlusion was probably related to secondary to reactive thrombocytosis after splenectomy. The patient was treated with steroids for papilledema and administered coumadin and aspirin. The symptoms resolved, and the findings returned to normal within three weeks. Current paper emphasizes that, besides other well-known thrombotic events, reactive thrombocytosis after splenectomy may cause central retinal vein occlusion, which may be the principal symptom of this risky complication. Thus, it can be concluded that followup for thrombocytosis and antithrombotic treatment, when necessary, are essential for these cases.

## 1. Introduction

Reactive thrombocytosis is usually known as a benign state that may occur after splenectomy and generally spontaneously resolves without thrombotic complications. However, further consequences may sometimes happen mostly in case of an extreme thrombosis in arterial or venous system [1].

Retinal vein occlusion, which is an obstruction of the retinal venous system, is the second most common sight-threatening retinal vascular disorder after diabetic retinopathy and may involve the central retinal vein or a branch retinal vein [2].

The aim of this study is to present a patient who developed acute central retinal vein occlusion secondary to reactive thrombocytosis after splenectomy and its treatment. To the best of our knowledge, no case of retinal vein occlusion associated reactive thrombocytosis after splenectomy was previously reported even though many cases associated with other hypercoagulability states were reported.

## 2. Case Presentation

A 26-year-old male patient was admitted to our department with the complaints of blurred vision and flashes of light, scintillating scotoma, and white spots in front of his right eye continuing for one week. The patient had a history of abdominal surgery for retroperitoneal paraganglioma, which was performed 2 months prior to the admission, and the procedure had included tumor excision and splenectomy. He stated that the postoperative period was uneventful and no further treatment including oncological agents had been offered after surgery.

**Figure 1:**
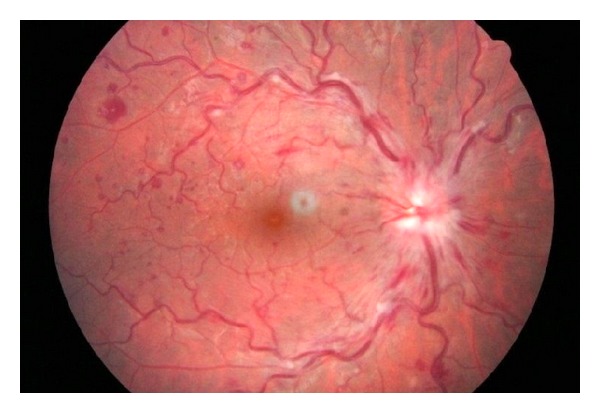
Scattered hemorrhages, increased venous tortuosity, and papilledema in the right eye.

**Figure 2:**
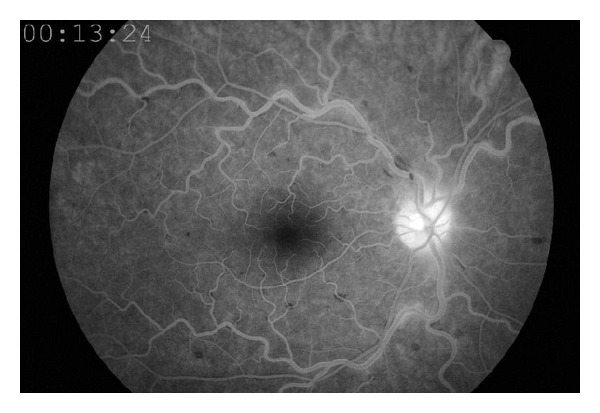
Fundus fluorescein angiography shows no ischemic signs.

**Figure 3:**
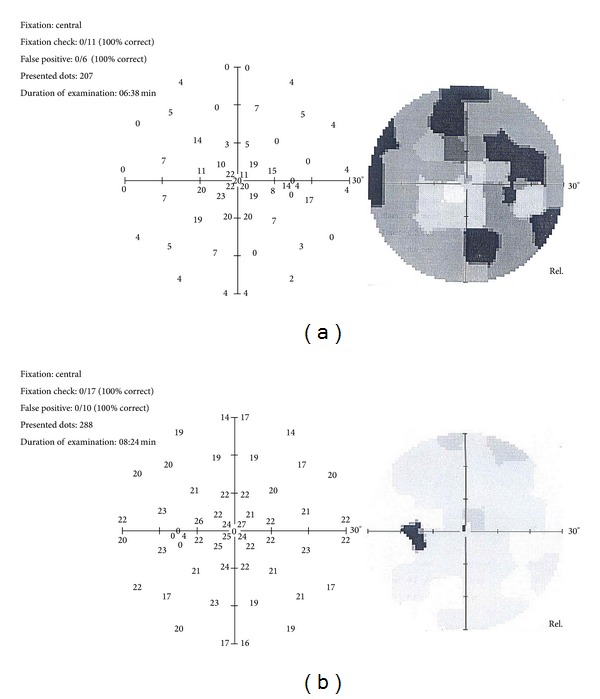
(a) Arcuate defects in the right eye's visual field examination. (b) Normal visual field findings in the left eye.

The ophthalmologic examination revealed that best corrected visual acuity was 20/20 and intraocular pressure was 12 mmHg in both eyes. Anterior segment examination revealed no pathologic changes. Fundus examination was normal in the left eye; however the fundus of right eye showed scattered retinal hemorrhages, increased venous tortuosity, and papilledema (Figure 1). Laboratory showed an elevated platelet count of up to 1,400,000/mL. Orbital and cranial MRI findings were normal. Fundus fluorescein angiography revealed a marked leakage on the optic nerve of the right eye, increased diameter, and tortuosity on veins without any sign of ischemia (Figure 2). Visual field evaluation by computerized perimeter was normal in left eye but there were arcuate visual field defects on right eye (Figure 3). The patient was hospitalized and treated by pulse dose steroid (1000 mg/day in four equal doses, which was converted to 80 mg daily on day 4) and oral coumadin (5 mg/day). He was discharged from the hospital with steroid (10 mg/day), aspirin (100 mg/day), and coumadin (arranged to achieve INR levels between 1.5 and 2.5) on day 8, when the platelet count dropped to 850.000. Weekly ophthalmological examination was continued for 2 months until it was totally normal, and the platelet count was 550.000/mL. Then, coumadin was stopped, but the patient was asked to continue oral aspirin (100 mg/day) and had no complaints or signs during the follow-up visit, which took place 3 months after the diagnosis.

## 3. Discussion

Reactive (secondary) thrombocytosis is characterized with elevated platelet count in response to infections, trauma, occult malignancy, surgery, myeloproliferative disorders, and splenectomy. The spleen plays a major role in platelet regulation since it is the primary destruction site of platelets. Accordingly thrombocytosis is reported to be observed in 75% to 82% of cases that have received a splenectomy. Platelet count generally returns to normal levels in weeks or months. This is usually a benign and self-limiting state but can result in thrombosis with an incidence of approximately 5% [3]. These thrombotic complications may affect both the venous and the arterial circulations. Arterial thrombotic events are primarily involving peripheral extremity and pulmonary, cerebral, and rarely myocardial arteries. On the other hand, venous thrombotic events include deep vein thrombosis, pulmonary embolism, portal and hepatic vein thrombosis, and retinal vein thrombosis [1, 4].

Retinal vein occlusion is one of the most frequent causes of painless loss of vision. The main conditions it is associated with include hypertension, diabetes mellitus, sedentary lifestyle, and open angle glaucoma, all of which are related to elder age. Other causes are smoking, drug consumption (i.e., contraceptives and diuretics), some inflammatory or immunologic diseases (i.e., syphilis, sarcoidosis, vasculitis, and HIV), and hypercoagulability conditions. It may be also caused by raised blood hemoglobin level and high plasma viscosity [2, 5].

Occlusion of the central retinal vein is clinically classified as ischemic and nonischemic types. The findings include multiple cotton wool spots, extensive retinal hemorrhage, and widespread capillary nonperfusion areas in ischemic type, whereas mild fundus changes, dot and blot hemorrhages, increased diameters of retinal veins, and papilledema are evident in nonischemic type. Two types may be differentiated with visual acuity and outcome, which are good in nonischemic types, but poor in ischemic types [6].

The patient, presented in the current report, had a nonischemic central retinal vein occlusion secondary to reactive thrombocytosis after splenectomy. Since he had not been given antithrombotic treatment after the initial operation, he had had extremely high platelet level. Since further investigation did not reveal any other possible reason for central retinal vein occlusion, we believe that reactive thrombocytosis was the cause of the disease. To the best of our knowledge, there are no previous reports in the literature describing reactive thrombocytosis as a cause of central retinal vein occlusion, although many cases with other hypercoagulability states have been reported. While the preventive effect of antithrombotic therapy has not been well-established in patients with retinal vein occlusion [7], we administered coumadin and aspirin treatment for monitoring of the platelet level. Although steroid therapy is controversial in cases with ischemic optic neuropathies [8], we also used steroids for the treatment of the patient owing to the fact that he had severe papilledema which may threaten visual prognosis. The patient quickly recovered during the follow-up period; ocular signs resolved and platelet levels returned to normal within weeks.

In conclusion, we believe that physicians should consider retinal vein occlusion besides other thrombotic events in cases with reactive thrombocytosis after splenectomy. In addition, current report underlines the importance of regular monitoring of the platelet count and thrombophylaxis when necessary after splenectomy, since these patients may present with central retinal vein occlusion besides other severe complications.
